# Clinical and diagnostic value of transcranial cerebral oximetry in the optimization of mechanical ventilation in newborn infants

**DOI:** 10.1186/cc9935

**Published:** 2011-03-11

**Authors:** V Estrin, A Simonova

**Affiliations:** 1 Research Institue of Obsetrics and Pediatrics, Rostov on Don, Russia

## Introduction

Treatment of ischemic damage to organs and tissues by mechanical ventilation with a high content of oxygen in the inspired mixture (FiO_2_) can lead to oxidative stress and reperfusion of tissue alteration, which is particularly characteristic of infants with their characteristic low levels of antioxidant protection. From this perspective, there is optimal mode selection in mechanical ventilation and FiO_2 _of vital organs and tissue, namely in brain tissue, which was made possible through the use of transcranial cerebral oximetry (TCO).

## Methods

At stage 1 of the study, with the consent of the ethics committee and informed parental consent, we examined 24 infants born in the physiological department of the maternity hospital RNIIAP of gestation 38 to 40 weeks, with Apgar 7 to 10, and birth weight 2,500 to 3,900 in the state of physiological sleep after feeding. In all children, we measured the cerebral tissue oxygen saturation (SctL, SctR) using the cerebral oximeter Fore-sight (USA) at 1, 3 and 5 days after birth. Later, in a controlled, randomized study were included two groups of neonates on mechanical ventilation. In patients of group 1 (*n *= 35), modes and ventilator FiO_2 _were determined under the control of TCO in a way that is as close as possible to indicators of cerebral oxygenation for the age norm. In patients of group 2 (*n *= 33), mode selection and FiO_2 _ventilation was carried out under the supervision of pulse oximetry and partial oxygen tension (pO_2_), according to acid-base balance, excluding indicators for TCO.

## Results

At stage 1 the study defined age-norm TCO indicators for healthy infants amounting in the left hemisphere of the brain to 79.2 ± 4.06% (0.01 <*P *< 0.05) and in the right hemisphere to 84.89 ± 5.1% (0.01 <*P *< 0.05). At phase 2 of the study group infants, the selection of modes and ventilator FiO_2 _on the basis of indicators for TCO statistically significantly (in all cases 0.01 <*P *< 0.05) decreased length of stay on the ventilator (from 9.4 to 5.6 bed-days), mortality (from 2.7% to 0%), and number of complications (cases of radiologically confirmed pneumonia from 4.2% to 0.2%) compared with the control group.

## Conclusions

The use of TCO for the optimization of mechanical ventilation and oxygen saturation monitoring in brain tissue in newborn infants in critical condition is a promising method for reducing mortality, reducing the term of ventilation and reducing complications of oxygen therapy in this group of patients.

